# Minimum Effective Volume of 0.75% Ropivacaine for Ultrasound-Guided Axillary Brachial Plexus Block

**DOI:** 10.7759/cureus.12229

**Published:** 2020-12-22

**Authors:** Koti Vadagandla, Vinay Jahagirdar, Kaanthi Rama, Danish Qavi

**Affiliations:** 1 Critical Care, Medicover Hospital, Hyderabad, IND; 2 Internal Medicine, Gandhi Medical College and Hospital, Secunderabad, IND; 3 Internal Medicine, Eras Lucknow Medical College, Lucknow, IND

**Keywords:** ultrasound guidance, axillary brachial plexus block, minimum effective volume, axillary block, brachial plexus block, nerve block, guided nerve block

## Abstract

Background

Ultrasound-guided peripheral nerve block provides direct visualization of nerve and reduces the complications associated with classical landmark guided technique, by reducing the dosage of local anesthetic drugs. This study aims to determine the minimum effective volume (MEAV) of 0.75% ropivacaine for ultrasound-guided axillary brachial plexus block.

Methodology

A total of 23 patients of age group 18-75 years belonging to ASA grade 1, 2, and 3 were selected based on inclusion criteria. The MEAV was determined by using Dixons & Massey Step-up and Step-down method. The initial volume was selected as 15 mL based on previous studies. Depending on block success or failure, 1 mL of the drug was decreased or increased. Block was assessed in terms of motor and sensory components. The study was aborted after attaining five cases of block failure, followed by five cases of a successful block.

Results

The MEAV to be given for a successful block in 50% of patients (MEAV50) was 8.62 mL (95%CI 3.54-9.89). The MEAV to be given for a successful block in 90% of patients (MEAV 90) was 11.82 mL (95% CI 9.9-75.7).

Conclusion

Ultrasound guidance reduces the dosage of local anesthetic drugs to be used and provides surgical anesthesia without any complications or adverse effects.

## Introduction

Axillary brachial plexus block is one of the most used peripheral nerve blocks for hand and forearm surgeries. This block is achieved by depositing the drug around the terminal branches of the brachial plexus in the axilla. Needle malposition is one of the main reasons for the failure of the block in the axillary region [1-3]. To provide a successful blockade by the classical landmark guided technique, a huge volume of the local anesthetic drug is required, the use of which is associated with complications such as cardiac dysrhythmias and tonic-clonic seizures [1-4]. Dose reduction may help reduce such complications [5,6]. Ultrasound guidance technique provides visualization of individual nerves and deposition of the drug around the nerve, which reduces the volume of the local anesthetic to be used [7-13].

## Materials and methods

This was a clinical, observational, hospital-based study, carried out over a period of one year in the Department of Anesthesiology at Bhagwan Mahaveer Jain Hospital, Bangalore, India. After ethics and scientific committee approval as per the institutional policy, all patients, who were scheduled to undergo elective upper limb surgery, were selected. Patients in the age group of 18 to 70 years, of both sexes, belonging to the American Society of Anesthesiology (ASA) grade 1, 2, and 3 were included in the study. The exclusion criteria included: Emergency surgeries, local site infection, bleeding coagulopathy/anticoagulants, and allergy to local anesthetics.

On the day before the surgery, a complete preoperative evaluation, with the necessary labs was done. Written informed consent was taken. Intravenous access was secured with an 18G cannula. Patients were monitored intraoperatively and placed on oxygen at 5 liters per minute via a facemask. Intravenous midazolam 1 mg was administered five minutes before the procedure.

Axillary brachial plexus block was performed using an ultrasound machine (Sonosite M Turbo), with an appropriate probe (8-12 MHZ, linear). The patients were in the supine position with the arm abducted and the elbow flexed to 90 degrees. After disinfection and skin antisepsis with chlorhexidine, the puncture site was infiltrated with 1% lidocaine. Axillary brachial plexus was identified and a starting dose of 15 mL of 0.75%. ropivacaine was injected, using a short beveled 22G insulated needle of 10 cm length.

The results of the block were analyzed in terms of sensory and motor block every 5 minutes up to 30 minutes. The time before administering the local anesthetic injection was considered as time zero. An observer who was not involved during the operation and oblivious to the anesthetic volume used, assessed the nerve blocks. The block was considered successful if the patient reached both the sensory block and motor block within 30 minutes of anesthetic administration. Otherwise, it was considered a failure. In case of a blockade failure, the amount of local anesthetic was increased by 1 mL for the next patient.

Motor block was assessed by the Modified Bromage scale (Table 1). A score of less than 2 was considered successful. Sensory block was assessed by lack of thermal sensitivity and response to pinprick in the regions of the median, ulnar, musculocutaneous, and radial nerves. Duration of the sensory blockade and motor blockade was assessed by asking the patient to record the time of first pain sensation and time of the return of motor power, respectively.

**Table 1:** Modified Bromage scale.

Grade	Definition
4	Full muscle strength in relevant muscle groups
3	Reduced strength, but able to move against resistance
2	Ability to move against gravity, but not against resistance
1	Discrete movements (trembling) of muscle groups
0	Lack of movement

Determination of MEAV50 and MEAV90

The determination of MEAV50 and its 95% confidence interval (CI) was based on the empirical formula of Dixon and Massey for large samples where the estimated MEAV50 is x = (∑fi xi / n) + d/2,

Where:

MEAV50 is the minimum effective volume of local anesthetic needed to produce an effective block in 50% of patients,

Xi is the local anesthetic (LA) volume used leading to a failed or successful block,

fi is the frequency of failed or successful blocks associated with Xi,

n is the total number of patients with failed or successful blocks, and

d is the volume interval.

The data were further analyzed using probit transformation and logistic regression to determine the effective volume of local anesthetic needed, to produce an effective block in 90% of patients (MEAV90).

Statistical analysis

For the calculation of the sample size, we assumed an SD (standard deviation) of 2.50 and an SEM (standard error of the mean) of 0.52 based on a previous literature review of local anesthetic doses [12,14]. The sample size required to measure ED50 (median effective dose) was derived by n = 2 (SD/SEM)2 as suggested by Dixon and Massey.

Nonparametric data were expressed as mean and SD. Categorical data were expressed as absolute and relative frequencies. Calculations were made in Medcalc and Graph Pad PRISMTM for Windows (Graph Pad Software Inc, San Diego, CA).

## Results

A total of 23 patients were selected based on inclusion criteria, between the age group of 18 years and 70 years. Among 23 patients, 17 were males and 6 were females. The type of surgical procedure performed is shown in Table 2. No patients were excluded from the study. The onset time for motor and sensory components for individual nerves are shown in Table 3. The study was terminated when there was a sequence of five cycles of failure or success.

**Table 2:** Type of surgical procedures performed. ORIF, open reduction and internal fixation.

Procedure	No. of patients	%
ORIF radius	3	13.0
Median nerve exploration	3	13.0
Forearm wound debridement	2	8.7
Tendon repair	2	8.7
Bursitis exploration	1	4.3
Cyst excision and debridement	1	4.3
Excision of dorsal ganglion	1	4.3
Extensor tendon repair	1	4.3
Finger reimplantation	1	4.3
Flexor tendon reconstruction	1	4.3
Ganglion excision	1	4.3
K wiring thumb and index finger	1	4.3
Middle finger tendon repair	1	4.3
Palmar fasciotomy	1	4.3
Ring finger tendon repair	1	4.3
Scar revision thumb	1	4.3
Wrist extensor tendon repair	1	4.3
Total	23	100.0

**Table 3:** Sensory and motor block onset time.

	Median nerve		Ulnar nerve		Radial nerve		Musculocutaneous nerve	
Onset time (min)	Motor	Sensory	Motor	Sensory	Motor	Sensory	Motor	Sensory
Mean	10	10.53	13.06	9.78	13.42	9.35	13.82	10
SD	3.34	5.24	3.04	5.53	4.43	5.07	4.85	7.07

**Figure 1:**
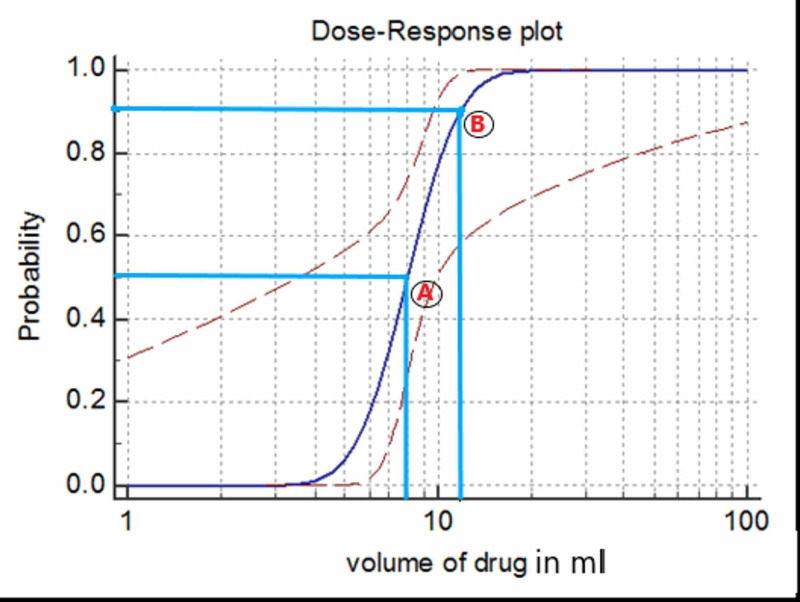
Logistic regression dose-response curve to estimate MEAV50 and MEAV90. A: Probability of successful block at 8.62 mL is 50% (MEAV50). B: Probability of successful block at 11.82 mL is 90% (MEAV90). MEAV: minimum effective volume.

The duration of blockade for motor and sensory components was 12.57±1.74 min and 9.9±0.49 min, respectively. MEAV50, calculated using Dixons and Massey empirical formula, was 8.625 mL (95% CI 3.54-9.89) and MEAV90, calculated with Probit transformation and logistic regression, was 11.82 mL (95%CI 9.9-75.7) as per the dose-response curve depicted in Figure 1.

There were no adverse events or inadvertent neuronal or vascular injuries. No additional analgesia or opioids were used during the procedure.

## Discussion

Regional blocks play a crucial role in modern-day anesthesia. Over time, the block technique has transformed from the classical, landmark guided, blind block practice to a more rational approach using ultrasonic guidance. Ultrasound allows a more accurate orientation of the nerve and eliminates potential complications such as inadvertent placement of needles and arterial puncture. This technique facilitates high precision viewing of the nerve structure and minimizes the dosage of the drug, thus avoiding unwanted systemic side effects of local anesthetic drugs.

Numerous studies have been conducted to evaluate the effectiveness of ultrasound guidance to reduce the MEAV of local anesthetic [12,14-16]. We conducted this study to assess the MEAV in the axillary brachial plexus block by ultrasound guidance with 0.75% ropivacaine.

In our study, the initial dose of 15 mL was arbitrarily selected based on a previous study by Ferraro et al, which also evaluated MEAV for an axillary brachial plexus block of individual nerves [17]. Due to considerable variation in the requirement of local anesthetic dose per nerve, we could not assess the block of individual nerve in our study. Many other studies conducted earlier to evaluate MEAV, also considered 15 mL as the starting dose of local anesthetic drug [15,17-19]. The methodology used to increase or decrease the dosage of the drug was based on Dixon and Massey step up and step-down method.

The mean onset time for the motor and sensory blockade in the current study was 12.57±1.74 min and 9.9±0.49 min respectively, with 0.75% ropivacaine for the axillary brachial plexus block. Philippe Gautier et al also used 0.75% ropivacaine for inter-scalene block and stated similar times of onset [8]. Gabrielle Lohom et al reported a shorter time for the onset of the block, which can be explained due to the rapid onset of action of 2% lignocaine with epinephrine, compared to ropivacaine [15].

0.75% ropivacaine was used due to its extended course of action and lower cardiotoxicity. The mean motor and sensory blockade return duration were 6.68±1.48 h and 8.78±1.74 h, respectively. Our result is similar to that obtained by Phillippe Gautier et al using 0.75% ropivacaine for interscalene block [8]. However, our results differ significantly from Lohom et al’s results in terms of duration of the block, which can be explained by the short duration of action for 2% lidocaine that they used, when compared to 0.75% ropivacaine [14].

The MEAV50 was 8.625 mL and MEAV90 was 11.82 mL and injected the local anesthetic into individual nerves of axillary brachial plexus [17]. They calculated the MEAV90 as 1.56 mL per nerve. By inserting a catheter into the interscalene nerve plexus, Vandepitte et al performed the block in 29 patients and observed that it takes less than 7 mL to achieve successful blockage in 12 patients [18]. But there are many limitations to their study, one being the displacement of catheters in consecutive patients, which gives variation in local anesthetic dose.

In our setting, we managed to do both superficial and deep structure procedures under the axillary brachial plexus block. O'Donnell and Gabrielle Lohom used axillary brachial plexus block to perform only superficial procedures of the forearm, which was a limitation in their study [15].

Congruent with other studies, our study demonstrates that the visualization of the entire nerve periphery and direct drug deposition helps in significantly decreasing the drug dosage and minimizes the complications related to the blind block technique [8,12,15,17,18,19,20]. The decrease in the dosage of local anesthetic can produce surgical anesthesia with the least complications. However, our study has certain limitations like limited number of patients covered (n = 23) which can restrict the data's external validity and use of ultrasound in primary and secondary health care settings is limited due to the high costs associated with the system and the need for qualified staff for proper and accurate techniques to provide brachial plexus block.

Two studies have different outcomes from ours. The study conducted by Duggan et al for supraclavicular block under ultrasound guidance technique failed to decrease the volume of anesthetic [14]. The possible reason for failure was mentioned as the requirement of larger volumes of the drug for plexus compared to individual nerves in axillary brachial plexus. In another study, Tran et al failed in achieving successful blockade due to certain limitations, including the use of the single needle technique in their study [16].

## Conclusions

The present study concludes that MEAV50 and MEAV90 for axillary brachial plexus block by ultrasound guidance technique was 8.6 mL (95%CI 3.5-9.8) and 11.8 mL respectively with 0.75% ropivacaine which is in concordance to other studies. By using the ultrasound guidance technique, it is possible to minimize the local anesthetic volume to achieve surgical anesthesia and analgesia, without any adverse effects and complications.
